# Introducing mobile apps to promote the well-being of German and Italian university students. A cross-national application of the Technology Acceptance Model

**DOI:** 10.1007/s12144-022-03856-8

**Published:** 2022-10-27

**Authors:** Giulia Paganin, Jennifer Apolinário-Hagen, Silvia Simbula

**Affiliations:** 1 Department of Medical and Surgical Science, Alma Mater Studiorum-University of Bologna, Bologna, 40126 Italy; 2 Institute of Occupational, Social and Environmental Medicine, Centre for Health and Society, Medical Faculty, Heinrich-Heine University Düsseldorf, Düsseldorf, Germany; 3 Department of Psychology, University of Milan-Bicocca, Milan, 20126 Italy; 4 Bicocca Center for Applied Psychology, Department of Psychology, University of Milan-Bicocca, Milan, 20126 Italy

**Keywords:** Structural invariance, Measurement invariance, Technology acceptance model, Cross-national studies, University student well-being

## Abstract

Stress represents a significant risk factor for several psychophysical diseases among college students, such as depression and anxiety, which may undermine their academic functioning, resulting in high drop rates from college. Nevertheless, university services for mental health promotion are typically underutilized. As a result, professionals and authorities strive to find new ways to address students' mental health needs. In this view, mobile apps seem appropriate for well-being promotion interventions. Drawing on the Technology Acceptance Model (TAM), which is the most widely used theory on users' intention to use technologies, we assumed that perceived usefulness (PU) and perceived ease of use (PEOU) would be positively related to intention to use (INT), and PEOU would be positively related to PU among both Italian and German university students. To test our hypotheses, we replicated the same cross-sectional study in Italy (*n* = 255) and Germany (*n* = 228) with university students. Although we found partial scalar invariance of the TAM dimensions across the two nations, our predictions were only partially confirmed: PEOU was positively related to PU in the Italian sample only. Overall, this study is one of the first empirical attempts to compare TAM cross-nationally within the European context and it contributes to the small but increasing body of research investigating students’ acceptance of smartphone-based interventions for stress management and well-being promotion. Understanding mobile health acceptance could help universities increase students’ chances of adopting the proposed services, considering the factors influencing this choice.

## Introduction

Recent scientific research reveals that university students are a risk population for mental health conditions (Baik et al., 2019). In this regard, it is estimated that more than 75% of college students aged 18 to 33 experience high-stress levels (Huberty et al., 2019). This is a topical issue for universities and the overall community. Given the widespread use of new technologies, including smartphones, mobile apps seem to be an appropriate solution to deliver well-being promotion and stress management interventions (Borghouts et al., 2021). Currently, professionals and authorities strive to find new ways to address students' mental health needs (Kern et al., 2018) since university mental health and well-being promotion face-to-face services are typically underutilized (Ponzo et al., 2020). This depends on several factors, such as long waiting lists, lack of time, fear of social stigma, negative treatment attitudes, the willingness to perform self-management, and perceived absence of needs (Amanvermez et al., 2020; Borghouts et al., 2021; Harrer et al., 2021; Kern et al., 2018). However, despite the widespread use of smartphone apps, the adoption rate of mental health apps is still relatively low, even among university students (Gaebel et al., 2021). Therefore, it is of the utmost importance to investigate the intentions to use mobile apps and better understand the factors that can influence them. The Technology Acceptance Model (TAM; Davis, 1989) is one of the most applied theoretical models to explain technology acceptance in several contexts (Sagnier et al., 2020; Yoon, 2016), even if the university students’ population are generally underrepresented (Borghouts et al., 2021). Moreover, even if different studies aim to understand the factors that could influence technology intention to use in several countries, such as the USA and China, only a few studies have adopted a cross-national perspective (Lo Presti et al., 2021; Yousafzai et al., 2007).

Therefore, the main goal of this study is to confirm the measurement invariance of the three-factor TAM scale among university students from Italy and Germany as two exemplary countries that are comparable in terms of cultural (European) background but differ in terms of their healthcare system and the implementation of digital mental health services. A further goal is to test whether the model analyzing the relationship between perceived ease of use, perceived usefulness, and intention to use would be structurally invariant across countries to understand better university students’ intention to use mobile apps for well-being promotion and stress management.

### Technology acceptance model

Research on technology acceptance has grown in importance since acceptance factors influence people's decision-making process in activities linked to the development, introduction, and usage of technologies (Orji, 2010). Indeed, according to the scientific literature, user approval is essential for developing any technology (Taherdoost, 2019). Technology adoption could be a huge barrier for those who create new technologies (Nadal et al., 2020). Therefore, it's crucial to comprehend the users' purpose for utilizing the innovation and their level of technological acceptability before bringing it to any setting (for example, a mobile app for university students' stress management and well-being promotion). Over the past 30 years, several theoretical models have been put out to analyze and explain the acceptance of and behaviors related to the introduction of technology. Particularly, the TAM (Davis, 1989) is the most widely used theoretical model for explaining technology intention to use (Rahimi et al., 2018), considering different types of technology (Drehlich et al., 2020), and users (Venkatesh et al., 2003), in a wide range of ICT application domains (Sagnier et al., 2020; Yoon, 2016). The TAM (Davis, 1989) was explicitly built on a consolidated theoretical model, the Theory of Reasoned Action (TRA; Fishbein, 1967; Fishbein & Ajzen, 1975). The TAM received excellent scientific support for their fundamental variables: Perceived Usefulness (PU), Perceived Ease of Use (PEOU), and Behavioral Intention to Use (INT). The TAM states that the intention to use a new technology depends mainly on the perceived usefulness (PU, i.e., the degree to which the technology is perceived as helpful to achieve one's goals) and the perceived ease of use of the technology (PEOU, i.e., the degree to which the technology is perceived as easy to use).

Moreover, the model indicates that PEOU impacts PU because, given the same functionalities and features, the more time a person can save using the more accessible technology, the more such technology is perceived as helpful to reach (faster) the intended goal (Venkatesh, 2000). TAM originally included attitude as an antecedent of INT, but it was removed to make the model more parsimonious (Gupta & Sahu, 2020). Some theoretical expansions have been created (e.g., UTAUT; Venkatesh et al., 2003), even if the core of these models has always remained the same. TAM has been used in various contexts, including the workplace. Recently, it has also been applied to other contexts wherein the use of technology is voluntary. Although there are numerous studies regarding technology acceptance in the educational context (Granić & Marangunić, 2019), empirical studies considering technology acceptance among university students represent a minority compared to research investigating this topic in other populations (Aboelmaged et al., 2021; Lee & Jung, 2018). In addition, only few studies have considered students' acceptance of mobile-app (e.g., Dederichs et al., 2021).

### Smartphone-based interventions

Stress and mental health problems among university students represent critical public health issues since healthy students will be the healthier employees of the future (Portoghese et al., 2019). Therefore, effective stress management programs have become a priority (Huberty et al., 2019) because institutions can help students prevent mental health issues and provide appropriate treatments (Amanvermez et al., 2020). As aforementioned, university students have access to multiple services but are typically underutilized (Ponzo et al., 2020). In addition to the obstacles listed in the previous paragraph, university students are likely to face several obstacles, despite recognizing that they require guidance. For instance, they may believe that their situation is common to all students and that their condition is due to their study load, becoming skeptical about the usefulness of available treatments (Lattie et al., 2019).

In recent decades, an increasing body of research has examined alternative ways to offer mental health and stress management interventions, with a recent shift from traditional face-to-face to smartphone-based interventions (Ryan et al., 2017). This has resulted in the definition of a novel domain, the so-called mobile Health (mHealth), defined as “medical and public health practice supported by mobile devices, such as mobile phones, patient monitoring devices, personal digital assistants (PDAs), and other wireless devices” (World Health Organization, 2011; p.6). Because of their properties, smartphones are well suited to provide mental health interventions. Smartphones allow researchers and professionals to monitor participants continuously and in a non-intrusive way, potentially reaching more people, maintaining anonymity, and customizing treatments based on their characteristics and needs (De Korte et al., 2018; Ryan et al., 2017). The literature points out that burnout, stress, despair, and anxiety may all be treated with mental health-tracking smartphone apps (Bregenzer et al., 2017; Carissoli et al., 2015; Meyer et al., 2018). Although the number of smartphone apps targeted at mental health has increased in recent decades, there is still a paucity of research on college students' acceptance of mental health apps (Kern et al., 2018). Indeed, most research has focused on the efficacy and acceptance of mental health apps in clinical settings. Therefore, little is known about which common factors could facilitate (versus hinder) stress management apps among university students and which specific factors could influence such use in particular sub-groups of this population (Kern et al., 2018).

### Differences between Germany and Italy

As stated before, this study aims to compare TAM cross-nationally among university students from Italy and Germany. Although they are both European countries, Italy and Germany differ in terms of their healthcare system, as well as in the implementation of digital mental health services, depending on some cultural differences**.**

#### The spread of digital interventions for mental health

Several European nations, such as Germany and Italy, provide public funds to pay the expenses of preventative healthcare. More specifically, based on the German Digital Healthcare Act passed in December 2019, German physicians and psychotherapists have the option to prescribe specific certified medical apps for treating and managing mental and somatic disorders (so-called 'apps for prescription') at the expense of statutory health insurance from October 2020. As of January 2022, 28 certified medical apps are listed in the DiGA directory (German, Digitale Gesundheitsanwendung; DiGA) of the Federal Institute for Drugs and Medical Devices (German, BfArM; https://diga.bfarm.de/de/verzeichnis). These apps are certified medical products with low risk for detecting, monitoring, managing or treating various diagnosed conditions such as depression, tinnitus, chronic pain, or cancer, including native apps and online platforms (web-based programs). In addition, in 2018, the German National Association of Statutory Health Insurance expanded its certification guidelines from traditional face-to-face to digitally supported health promotion and primary prevention programs using information and communication technologies to increase utilization rates and access to primary prevention (e.g., by using stress management apps; Apolinário-Hagen et al., 2020).

On the other hand, interest in telemedicine has only recently arisen in Italy. In 2012, the Italian Ministry of Health published national guidelines to frame and regulate this nascent medicine application to health promotion and care. Only with the advent of the Covid-19-pandemic a new document was approved on 17 December 2020 between the Italian government and regions, titled 'National Guidelines for the Provision of Telemedicine Services. As a result, health services through telemedicine have officially become part of the opportunities the National Health Service offers. The document provides the indications adopted at the national level to provide certain telemedicine services, such as telehealth, medical teleconsultation, tele-assistance by the health professions, and tele-referral.

#### Cultural differences

Culture may explain national, corporate and group behavioral patterns or attitudes. It can also impact how successfully technology is implemented and used (Leidner & Kayworth, 2006). According to Hofstede (1984), culture is "a communal programming of the mind." Culture, often referred to as a national character, has been defined as the patterns of personality traits shared by citizens of the same country (Clark, 1990). Considering this, it is crucial to understand the culture in order to research information technologies, particularly in the context of new technologies.

Based on previous work, Hofstede (1984) analyzed the cultural differences of workers from numerous countries concerning four key dimensions: power distance, individualism (vs collectivism), masculinity (vs femininity) and uncertainty avoidance. Referring to the analysis carried out in previous work (see Leimeister et al., 2009), we can see that Italy and Germany share scores on three out of four dimensions, except for that Uncertainty Avoidance. Germany scores low on this dimension, suggesting that Germans are highly motivated to achieve goals, even by taking risks. In contrast, Italians score higher on this dimension, signifying that they are more reluctant to take risks to achieve goals. Concerning technology, we can say that those who score lower are more inclined to try new technologies, to face new situations. Although several empirical attempts have been conducted to analyze the factors that could influence technology's intention to use in several non-European countries (e.g., the USA and China), only a few studies have considered the European context (Lo Presti et al., 2021; Yousafzai et al., 2007), using a cross-national comparative perspective (e.g., Nistor et al., 2013). Therefore, we are interested in investigating whether there is a difference concerning the intention to use technology between university students from these two countries, considering the cultural differences between Italians and Germans.

### Objective and Hypotheses

Although studies on technology acceptance in students are increasing, to the best of our knowledge, there is not a single study investigating this topic using a cross-national comparative perspective between Germany and Italy. Moreover, more research is needed to examine the acceptance of specific forms of technology (e.g., mobile apps) to make the TAM theoretical model even stronger (Teo & Zhou, 2014). Therefore, we tested the TAM model of measurement across the two nations. We expect that the Italian and German versions of the TAM scale will show a three-factor measurement model. A further goal was to examine whether the factor structure of the TAM scale and the structural model analyzing the relationship between PEOU, PU and INT would be structurally invariant across countries.

These objectives led to the formulation of the following hypotheses:

- H1. The technology acceptance measure will present a three-factor structure in both the German and Italian samples;
- H2. The technology acceptance measure will present measurement invariance between Italy and Germany;
- H3. The technology acceptance model will present a structural invariance between Italy and Germany: Students’ intention to use the mobile app will be directly influenced by PU (H3a) and PEOU (H3b), and PU will be directly related to PEOU (H3c).

## Materials and methods

### Participants and recruitment

Data collections were conducted in Italy and Germany between July and Decembe,r and involved university students enrolled in academic degree courses. German participants were recruited via Prolific^1^ and social media posts. Prolific is a newly launched online participant recruitment platform that helps researchers recruit niche or representative samples on demand. Prolific combines high recruiting criteria with low costs, and participants are explicitly informed that they are being recruited for the study (Palan & Schitter, 2018). German participants were also recruited using social media platforms (e.g., Facebook, Instagram) and posts on the websites of two German institutes (Institute of Occupational, Social and Environmental Medicine at the Heinrich Heine University Düsseldorf and the Department of Health Psychology at the University of Hagen), and a section for study recruitment of the German website of Psychology Today (German, “Psychologie Heute”). Italian participants were recruited via social media platforms (e.g., Facebook, Instagram, WhatsApp) and recruitment platforms of the Department of Psychology of the University of Milano-Bicocca. The questionnaire was disseminated via the Qualtrics link. The data collection was carried out following the Declaration of Helsinki's ethical norms, and the Ethical Committee of the Department of Psychology of the University of Milano-Bicocca approved the research (Prot. N. RM-2020–312). Italian and German participants were informed about the study’s goals, the complete voluntariness of their participation, and the possibility of withdrawing from the study at any time. If research participants had any questions, they could use the email contact of one of the researchers to obtain clarifications. Moreover, before starting the questionnaire, participants were required to read the informed consent forms and provide their anonymous informed consent. In order to ensure that each participant had a clear understanding of mobile apps for promoting well-being and stress management, respondents were given a detailed description of the functionalities and aims of WellBe!, a smartphone app developed by the Bicocca Center for Applied Psychology (BiCApP). This specific app was chosen for two main reasons. The first was not to run into copyright issues, which could occur using an app that already existed on the market; the second was to put the participants in a position to reflect on the use of an app that no one had experience with, which could have created a bias between the responses of Italian and German students. WellBe! was developed based on Positive Psychology concepts. This app intends to assist users in developing personal resources and skills to help them manage their stress. WellBe! allows users to track their stress levels and intervene in a tailored way to improve their mental health. WellBe! suggests daily self-help exercises based on proven scientific studies. The final convenience sample was composed of 483 participants. The Italian sample was composed of 255 respondents. Most respondents were female (85.90%) with an average age of 23 years (SD = 4.06) and were enrolled in a humanistic degree course (94.4%). On the other hand, the German sample was composed of 228 respondents. Most respondents were female (66.20%) with an average age of 24.48 years (SD = 8.63) and were enrolled in a humanistic degree course (63.2%, followed by 34.2% enrolled in a scientific or technical degree course). In both samples, all participants were familiar with using smartphones. Regarding previous experience with well-being smartphone apps, 14,5% of the Italian students and 37.3% of the German students reported experience with such apps. Chi-square analysis showed there were statistically significant differences between Italian and German students regarding previous app experiences (χ2 (1, *N* = 483) = 33,061, *p* = 0.001). To better describe our sample, we calculated the ratio between effort and reward perceived by Italian and German students. Both samples do not report an imbalance between the effort required by their student work and the rewards awarded (Italian students: M = 1.02, SD = 0.32; German students: M = 0.83, SD = 0.43).

### Measures

Regarding TAM dimensions, we used three items to evaluate perceived ease of use (e.g., “It will be simple to use WellBe!”), four items to assess perceived usefulness (e.g., “WellBe! could help me improve my well-being”), and three items to measure intention to use (e.g., “I would like to try WellBe!”). The items were graded on a five-point Likert scale, where 1 indicates “strong disagreement” and 5 indicates “strong agreement”. The items were utilized in prior published scientific studies (Apolinário-Hagen et al., 2019; Curcuruto et al., 2009; Davis, 1989; Paganin & Simbula, 2021) and already translated using back-translation techniques (Brislin, 1970). In order to get more information about the sample, we employed the Effort-Reward Imbalance Questionnaire-student version (Italian version by Portoghese et al., 2019). The scale is composed of two subscales: effort (e.g., constant time pressure due to a heavy study load) and reward (e.g., I receive the respect I deserve from my fellow students). The subscales are composed respectively of three and six items; both subscales are assessed using a 4-point Likert scale, ranking from 1 (strongly disagree) to 4 (strongly agree). To determine the ER ratio, we place the effort score in the numerator and the reward score in the denominator with the following formula: ER = E R*c. Specifically, the E is the effort rating, R is the reward rating, and c is a correction factor that accounts for the effort and reward scores' uneven number of items. In order to interpret the ER-Ratio, the authors suggest that when ER = 1, the person reports just one effort for each reward; when ER 1, the person reports fewer efforts for each reward; and when ER > 1, the person reports more efforts for each reward (Siegrist et al. 2014). In both the Italian and German samples, all scales revealed good reliability, with Cronbach’s alpha values higher than 0.80 (i.e., the generally accepted standard; Taber, 2018), except for the ERI scale. In particular, in the Italian subsample, the effort dimension showed an alpha value of 0.50. To obtain a more acceptable reliability value, we followed what was done in the article by Portoghese et al. (2019). As already highlighted in the validation study of the scale by Wege et al. (2017), we eliminated item 2 of the "effort" dimension. The new alpha value was 0.60 in the Italian and 0.70 in the German subsample.

### Statistical analysis

Using SPSS 27, descriptive statistics of items were analyzed to establish the normality of the data and the robustness of subsequent analyses (George & Mallery, 2016). Multivariate outliers were identified using the p < 0.001 criterion for Mahalanobis distance, and statistical assumptions (Kaiser–Meyer–Olkin measure and Bartlett test groups) were validated. Firstly, two confirmatory factor analyses (CFAs) were conducted separately for Italian and German students. Next, to evaluate measurement invariance across nations, we conducted a series of multiple-group confirmatory factor analyses (MGCFAs) with the robust maximum likelihood with robust standard errors (MLR) method using Mplus 7 (Cheung, 2008). To estimate the model goodness of fit, we considered the following values: Comparative Fit Index (*CFI*, Bentler, 1990; values above 0.90 are usually considered to be indicative of a good model fit), Tucker-Lewis index (*TLI*, Tucker & Lewis, 1973; values above 0.90 are generally considered to be indicative of a good model fit), Root Mean Squared Error of Approximation (*RMSEA*, Steiger, 1990); values less than 0.08 and 0.05 suggest an adequate and good model fit) and Standardized Root Mean Square Residual (*SRMR*; values of 0.05 are taken as a good fit, 0.05-0.07 as moderate fit; Brown, 2015). In order to detect statistical differences between models, the χ2 of the baseline model was subtracted from the χ2 value of the nested comparison model, obtaining the Satorra-Bentler scaled χ2 (Satorra & Bentler, 2010).

Moreover, to test the between-group invariance of CFA models, we computed the difference in CFIs between the freely estimated model and the constrained model supporting invariance when this value was up to 0.10 or below (Meade et al., 2008). The MGCFAs were conducted in the following order analyzing: a) configural invariance that requires only the same number and pattern of factor loadings, not necessarily equivalent factor loadings, are the same across groups; b) metric invariance that requires that factors loadings are identical between the two groups while ensuring the factor variances and covariances are free to vary; c) strong factorial invariance that requires that factors loadings and mean intercepts are identical between groups; d) strict factorial invariance that requires that factors loadings, mean intercepts and unique variances are identical between groups. Since we could not reach full strict invariance, we tested for partial strict invariance by removing some equality constraints from unique variances across groups. In order to be able to compare results between two groups, it is necessary to obtain at least the scalar invariance of the measurement (Fischer & Karl, 2019). Then, we verified whether we could confirm the structural invariance of the TAM by introducing one invariant path at a time and then computing the difference between the baseline model’ and constraint models χ2.

### Results

Skewness and kurtosis indexes showed a normal distribution of the items (values ranging from -0.87 to 0.13 for skewness and values ranging from -0.80 to 0.69 for kurtosis). An examination of the Mahalanobis distance scores indicated the presence of one multivariate outlier that was removed. The Barlett’s Test of Sphericity was significant (*p* = 0.000), and the Kaiser–Meyer–Olkin measure was satisfactory (0.86).

To confirm the psychometric validity of the three-factor TAM measure (H1), two independent CFAs were conducted in the two sub-samples (Italy and Germany). We used the above-mentioned three-factor model: PU, PEOU, and INT. The results (see Table 1) indicated that the model adequately fit the data in both the Italian (χ2(32) = 45.70, CFI = 0.99, RMSEA [90% CI] = 0.041 [0.00, 0.06], SRMR = 0.021) and German samples (χ2(32) = 43.66, CFI = 0.99, RMSEA [90% CI] = 0.04 [0.05, 0.07], SRMR = 0.03). Cronbach’s alphas varied from α = 0.88 to α = 0.94 in the Italian sub-sample and ranged from α = 0.861 to α = 0.93 in the German sub-sample. Then, we run four MGCFAs. Results for the configural model CFA indicated a good model fit (see Table 1), reflecting that the three-factor model and the factor pattern loadings were equivalent across nations. To this aim, the second CFA assessed the equality of factor loadings. Factor loadings were constrained to be equal across the two groups. We found a statistically non-significant difference in the χ^*2*^ statistic between the configural and metric factorial invariance models (Δχ ^2^ = 6.91, Δdf = 7) and the difference in CFIs was below 0.01 (ΔCFI = 0.000). Then, we tested the scalar invariance (or equivalence of item intercepts), constraining the item intercepts to be equal in the two groups and retaining the constraints applied in the previous model. In this case, we obtained a significant χ^*2*^ statistic difference (Δχ ^2^ = 43.42, Δdf = 9). As a result, full scalar invariance could not be demonstrated. However, partial scalar invariance can still be demonstrated (Millsap & Meredith, 2007). To this end, the non-invariance of the intercepts was explored by releasing constraints on the intercepts one by one based on modification indices. Item 1 was relaxed for reaching partial scalar invariance. The difference in the χ^*2*^ statistic for the metric factorial invariance and the partial scalar invariance (where the intercept of item 1, belonging to the PU dimension, was freely estimated) models was not statistically significant (Δχ ^2^ = 13.91, Δdf = 7), and the difference in CFIs was below 0.01 (ΔCFI = 0.006). Hence, since partial scalar invariance was supported, the partial residual variance was tested by constraining all item residuals except item 1 to be equivalent in the two groups. The difference in the χ^*2*^ statistic for the partial scalar invariance and the partial residual invariance models was not statistically significant (Δχ ^2^ = 14.76, Δdf = 1), and the difference in CFIs was below 0.01 (ΔCFI = 0.007). Therefore, partial residual invariance was confirmed. Since at least partial scalar invariance was confirmed, the TAM dimensions' scores were comparable across nations (Milfont & Fischer, 2010).

**Table 1:** MGCFA results for measurement invariance across the nation (overall sample, *N* = 483)

Model	χ^2^	df	Δ χ ^2^	Δdf	*p*	CFI	RMSEA	90% CI RMSEA	ΔCFI
Italian sub-sample	45.70	32	–	–	0.38	0.994	0.04	[0.00,0.06]	–
German sub-sample	43.66	32	–	–	0.05	0.993	0.04	[0.05,0.07]	–
Configural invariance	66.83	64	–	–	0.38	0.999	0.01	[0.00,0.04]	
Metric invariance	73.838	71	6.91	7	0.39	0.999	0.01	[0.00,0.04]	0.000
Scalar invariance	113.121	81	43.42	9	0.01	0.930	0.08	[0.06,0.10]	0.046
Part. scalar invariance^a^	99.66	80	14.76	1	0.07	0.992	0.03	[0.00,0.05]	0.007
Part. residual invariance	109.15	89	9.24	7	0.24	0.968	0.05	[0.03,0.07]	0.002

^a^Partial invariance was reached, freeing the intercept of item 1 “*WellBe! could help me improve my well-being*”

To also evaluate the comparability of the structural model of TAM, we compared an entirely freely estimated model to a series of even more constrained models. To test the invariance of the structural model, we adopted a stepwise strategy, testing a model with one path set equal in the two subsamples at a time. This procedure allowed us to identify a different pattern between the Italian and German subsamples.

We confirmed the invariance of both the direct path from perceived usefulness to intention to use (H3a) and the direct path from perceived ease of use to intention to use (H3b), but we did not confirm the invariance of the path from perceived ease of use to perceived usefulness (H3c; see Table 2).

**Table 2:** Testing for structural invariance of the Technology Acceptance Model

Model	χ^2^	df	Δ χ ^2^	Δdf	CFI	RMSEA	90% CI RMSEA	ΔCFI
Completely free model	118.475	89		–	0.987	.038	[.02,.06]	–
PU → INT	119.012	90	0.537	1	0.988	.037	[.02,.06]	.001
PEOU → INT	119.956	91	0.944	1	0.988	.038	[.02,.06]	.000
PEOU → PU	126.153	92	6.197	1	0.986	.040	[.02,.06]	.004

*PU* perceived usefulness, *PEOU* perceived ease of use, *INT* intention of use

**Fig. 1:**
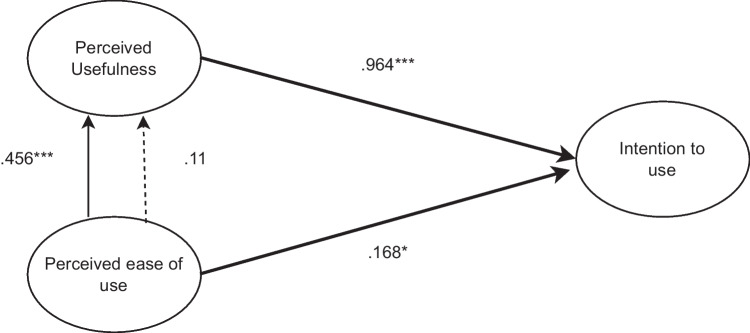
Technology acceptance structural model

Indeed, in the Italian subsample, we found a direct effect of perceived ease of use on perceived usefulness (β = 0.46; *p* < 0.001), while in the German subsample, we could not confirm the direct impact of perceived ease of use on perceived usefulness (β = 0.11; *p* = 0.218; see Fig. 1).

### Discussion

Our study aimed to confirm that the three-factor structure of the TAM scale and the structural Technology Acceptance Model were invariant across countries. We confirm our first hypothesis (H1): the TAM three-factor structure was invariant across nations. This finding provides further evidence that TAM is composed of Perceived Usefulness, Perceived Ease of Use and Intention to Use, which is in line with what was proposed initially (Davis, 1989).

Regarding our second hypothesis (H2), our results did not support full scalar invariance but only partial scalar invariance across nations, given the existence of one non-invariant item intercept of the PU dimension, meaning that this item had different connotations for Italian than for German students. This item refers to the perceived usefulness of WellBe! in promoting well-being. A possible explanation for this difference might be dissimilarity in beliefs about the usefulness of technologies among nations. Taking the cultural dimensions studied by Hofstede (1984) into account, previous studies (Leimeister, et al., 2009) demonstrated that people located in Italy versus Germany differ on the Uncertainty Avoidance cultural dimension. Considering these previous results, we can speculate that university students from the German sample might have a low score on this dimension compared to the students from the Italian sample, who might show a higher Uncertainty Avoidance. We can also suppose that the students from Germany are highly motivated to achieve their goals, even taking risks. In contrast, Italians that score higher on this dimension, could be more reluctant to take risks to achieve their goals. Taking into account these assumptions in the context of new technology introduction, such as apps for stress management and the promotion of well-being, we can say that those with a lower score (in our study, German students) are more inclined to try new technologies to face new situations. Thus, given the broader spread of mobile apps in Germany than in Italy, together with the different inclusion in the national health agenda, German students might be more confident that a high-quality mobile app for mental health can help them to improve their well-being than Italian students. The latter, who live in a nation where apps are still not so widespread in the healthcare context or within universities, may be less likely to believe that an app for mental health can be helpful to improve their well-being. Except for this item, our results indicate that the TAM scale can be used across the two nations.

We also partially confirmed the structural model invariance regarding the third hypothesis (H3). We can confirm the direct effect of PU on PEOU (H1a) and of PEOU on INT. As shown by several previous studies, it is crucial to increase individuals’ intention to use a specific technology in order to make them perceive such technology as beneficial (e.g., fundamental in achieving one’s purpose) and easy to use (e.g., that permit not to waste one's own time to accomplish the considered goal). In this case, it is vital to increase Italian and German students’ intentions to use the app WellBe! to convince them that it is easy to use and helpful to achieving their well-being and managing their stress. Moreover, our results show that, in both countries, perceived usefulness had a more significant effect on the intention to use compared to perceived ease of use, which is in line with the TAM hypothesis (Davis, 1989). However, we could not confirm our last hypothesis (H4c): PEOU was directly related to perceived usefulness only in the Italian sample. These results contrast with the traditional TAM literature stating that individuals are more likely to perceive technology as useful when they believe it is easy to use and advantageous to them in terms of time and resources to reach their proposed health goals. Only a few studies did not confirm this relationship. For example, a study by Lee & Lehto (2013) found that perceived ease of use did not consistently affect users’ acceptance decisions. A possible explanation for this cross-national difference might be related to the different mobile-app distribution and availability. In Italy, where students do not widely use mobile apps for mental health, perceptions of mobile apps as easy to use could still influence the extent to which they perceive the app's usefulness in terms of gain of time for their well-being. For example, one of the main barriers to accessing university mental health services is a lack of time (Amanvermez et al., 2020). Using an app for promoting one’s well-being could help to save some time compared to other services.

Conversely, in Germany, where mobile apps for mental health are more widely spread than in Italy, the extent to which students perceive that the app is easy to use is still important (see the direct effect on the intention to use it). Still, it is not so influential in changing the perceived usefulness of the app, which may be affected by other variables. A qualitative study on German medical students showed that although they were aware of the ease of use and flexibility of mental health promotion apps, these advantages did not make these tools ‘better’ than traditional interventions. Said differently, the app’s ease of use did not influence its perceived usefulness.

Furthermore, the same study found that participants were more likely to utilize a mobile app suggested by their institution (Dederichs et al., 2021). In our case, Wellbe! was presented and designed by an Italian institution, which could have influenced their opinion. Finally, previous studies showed a link between perceived stress and acceptance of mobile app (e.g., Ervasti et al., 2019). Our German subsample showed a slight imbalance in favor of the rewards obtained for one's efforts in studying. This might have influenced the German students' perception of the app's usefulness: in this case, they were not in a situation of need from the point of view of perceived stress, although they found the WellBe! app valuable and easy to use per se, the ease of use did not become an added value to increase the perceived usefulness.

Moreover, some studies stressed the importance of the “conditional value” in users’ behaviors. For example, Lee et al. (2017) refer to the concept of "conditional value", namely "a value existing in a specific context derived from circumstances in which the person is worried about his/her health" (Lee et al., 2017, pg. 231). In our case, as suggested by the E-I ratio, the students probably did not perceive any stress-related worries regarding their health. Specifically, both samples did not report an imbalance between the effort required by the students’ work and the rewards assigned. However, it should be noted that in both samples, the percentage of students who had already used apps to promote well-being is low, although the number of German students with such experience is higher than Italian students. This underlines that there is still a long way to go concerning the dissemination of such interventions, which are still little known, and the benefits are not yet well illustrated.

### Theoretical and practical implication

In the last decade, there has been an increase in the negative mental health symptoms reported by university students, suggesting a progressive worsening of their health status (Lattie et al., 2019). This could also be exacerbated by the advent of the COVID-19 pandemic (Liu et al., 2020). Thus, finding a viable option for delivering effective mental health interventions is crucial. In this regard, smartphone-based interventions are promising because of their potential to reach a broad plethora of students while at the same time avoiding some of the most commonly experienced barriers, such as fear of social stigma, predisposition to perform self-management, or apparent absence of needs (Amanvermez et al., 2020; Harrer et al., 2021). In order to design effective smartphone-based interventions for mental health, it is crucial to understand which factors influence technology acceptance among university students before introducing these technologies on a large scale. At the same time, it is essential to understand whether the theoretical construct of technology acceptance is structured equally across and perceived similarly by students from different European countries.

The present study has some theoretical and practical implications. Firstly, several cross-cultural studies in the literature have looked at the variables of TAM in different cultural contexts. However, to the best of our knowledge, very few studies to date have considered Italy, and none have considered a population of university students. From this point of view, the present study confirmed measurement invariance between Italy and Germany in a sample of students, laying the foundation for using such instruments before the introduction of new smartphone-based interventions for stress management. Secondly, although TAM has been used in numerous contexts, targeting different users and technologies, there are very few studies on the acceptance of smartphone-based stress management interventions. Moreover, the population of university students was only marginally considered. Therefore, to date, the present study is one of the few that has regarded the intention to use mHealth tools for stress management in university students.

With regard to the practical implications, several studies have emphasized the importance of questioning users before introducing new technological tools. Most of the studies, however, were carried out in organizational contexts. The present study has the merit of validating a tool applicable to the university context. Moreover, although the TAM has been used with various technologies, as previously pointed out, very few studies consider the intention to use mHealth tools in a historical moment that, for multiple reasons, pushes for the digitization of interventions for the promotion of health, including mental health. This study, therefore, provides a flexible and usable tool to assess the intention to use smartphone-based interventions. Such tool can be used together with or instead of traditional face-to-face interventions to improve university students’ well-being. Finally, even if the percentage of stress management apps usage was low in both samples, German students, who present a higher rate in terms of experience, present a slightly different acceptance pattern, in particular regarding the influence of PEOU on PU, compared to the Italian ones. This demonstrates the importance of also considering prior experience, as well as assessing user acceptance before new technologies are introduced.

### Limits and future direction

These findings should be interpreted in light of some limitations. First, our findings are limited in generalizability to two European countries which share values and cultural backgrounds. Thus, future investigations should replicate these results in other nations with different cultural configurations to increase the generalizability of our results. Second, other variables we did not directly measure could have influenced students' technology acceptance. Then, future work should consider other factors influencing technology acceptance to deepen our understanding of the underlying reasons for technology adoption. Third, this cross-sectional study merely relied on self-report measures. Thus, future studies should integrate self-report measures with objective indicators (e.g., actual use), collect data from multiple sources, and adopt a longitudinal design. The risk is having excellent theoretical insights which do not apply to actual behavior. University students' acceptance of technology for their mental health is growing, and there is a strong need to create a theoretical basis to better tailor technology introduction in academia. Our results also suggest several future research perspectives. On the one hand, further studies should replicate our results in European, American, and Asian countries to identify the different acceptance patterns among students. On the other hand, more research is needed to deepen the understanding of acceptance and investigate the intrinsic motivations that push students to adopt mobile-based interventions. Understanding which app features and functionalities might influence students' intentions to adopt smartphone-based interventions is also essential. Finally, establishing the role of previous experiences in promoting the intention to use new technologies (see, for example, Venkatesh et al., 2012) could be fundamental to replicating the current research in a few years, when also in the Italian context, the smartphone-based intervention will be well-known and more broadly adopted.

## Conclusion

This study contributes to understanding factors influencing university students' adoption of apps for well-being promotion and stress management interventions. Over the past decades, we have witnessed increased mental health issues among university students, further increasing after the COVID-19 pandemic. This health emergency, which has affected every country in the world indiscriminately, has stressed the need to implement new services to promote students’ psychophysical well-being, replacing those that already exist and are generally underused. Due to their non-intrusive characteristics, smartphone-based interventions can reach a wide range of especially young audiences while maintaining anonymity.

The current study allowed us to confirm the invariance of the scales used to investigate the acceptance of the technology in two different countries, Italy and Germany. Besides establishing the measurement invariance, it was possible to confirm the structural model's invariance only partially. Our results showed that the perception of ease of use and usefulness impact the intention to use. These results may help institutions and universities design awareness campaigns to encourage students to use apps to promote their well-being. It is important to emphasize that mobile apps can guarantee the anonymity of participants, thus limiting students' fear and perception of social stigma; they are also generally easy to use and can be less time-consuming than face-to-face interventions, allowing students to focus on other things (e.g., studying and preparing for exams). However, there seems to be a difference in perceived user-friendliness between Italy and Germany, which suggests that other elements may influence this relationship. Although participants in recent research are accustomed to using technology, there is still a widespread distrust of the effectiveness of smartphone-based interventions (e.g., Dederichs et al., 2021) and their subsequent adoption. The next step would be to identify the causes of this mistrust by analyzing the acceptance of students from different university faculties to highlight possibly different motivations.

## Data Availability

The datasets generated during and/or analyzed during the current study are available from the corresponding author on reasonable request.

## Footnotes

1 https://prolific.co/

## Declarations

All procedures performed in studies involving human participants were in accordance with the ethical standards and with the Helsinki Declaration and its later amendments or comparable ethical standards.
